# Implementation of an antimicrobial stewardship program in the Australian private hospital system: qualitative study of attitudes to antimicrobial resistance and antimicrobial stewardship

**DOI:** 10.1186/s12913-022-08938-8

**Published:** 2022-12-20

**Authors:** Darshini Ayton, Eliza Watson, Juliana M. Betts, Joseph Doyle, Benjamin Teh, Glenn Valoppi, Menino Cotta, Megan Robertson, Trisha Peel

**Affiliations:** 1 Health and Social Care Unit, School of Public Health and Preventive Medicine, Monash University, Melbourne, VIC Australia; 2 Department of Epidemiology and Preventive Medicine, Monash University, Melbourne, VIC Australia; 3 Department of Infectious Diseases, Alfred Hospital and Central Clinical School, Monash University, Level 2, 85 Commercial Road, VIC Melbourne, 3004 Australia; 4 Epworth HealthCare, Melbourne, VIC Australia; 5 Department of Infectious Diseases, Peter MacCallum Cancer Centre, Melbourne, Australia; 6 Sir Peter MacCallum Department of Oncology, University of Melbourne, Melbourne, VIC Australia; 7 Slade Pharmacy, Melbourne, VIC Australia; 8 UQCCR, Faculty of Medicine, The University of Queensland, Brisbane, QLD Australia

**Keywords:** Antimicrobial Stewardship, Program evaluation, Private healthcare facility

## Abstract

**Background:**

Antimicrobial Stewardship (AMS) is a key method to tackle antimicrobial resistance (AMR). In Australia, private hospitals have a higher rate of inappropriate prescribing and non-compliance with antimicrobial guidelines, yet this phenomenon is poorly described. Private hospitals make up 49% of hospitals in Australia, making it vital to understand AMS in this setting.

**Methods:**

This study aimed to explore capabilities, opportunities and motivations for AMR and AMS with stakeholders at an Australian private hospital, and identify barriers and enablers 5 years post-implementation of an AMS program comparing with pre-implementation findings. A mixed-methods study was performed, involving three focus groups with stakeholders. All doctors, nurses and pharmacists at the hospital were invited to complete a survey on their experiences with and awareness of AMR, AMS and antimicrobial prescribing.

**Results:**

Thirteen staff took part in the focus groups, 100 staff responded to the survey. Staff understood the importance of the AMS program, but active engagement was low. Staff felt more thorough feedback and monitoring could improve prescribing behaviour, but acknowledged difficulty in private hospitals in changing habits of staff who valued autonomy in making prescribing decisions. Half of respondents felt the current AMS restrictions should continue. Executive engagement may be needed to drive system changes across a complex network.

**Conclusion:**

AMS awareness increased post-implementation, but staff remained sceptical of its benefits. Engagement and education of medical consultants regarding local benefits of AMS must improve. Enhanced understanding of feedback provision, methods for engagement, and advocacy from leadership will ensure success and longevity for the program.

**Supplementary Information:**

The online version contains supplementary material available at 10.1186/s12913-022-08938-8.

## Background

Antimicrobial resistance (AMR) is a global health problem which threatens to undermine the significant health gains of the past century. A major contributing factor to AMR is inappropriate prescription and use of antimicrobials in hospital and community settings [1]. The World Health Organization (WHO) and the Australian Government have highlighted the importance of Antimicrobial Stewardship (AMS) programs as one mechanism for addressing this issue [1, 2]. AMS programs involve a combination of restrictive and enabling strategies to achieve more appropriate prescribing [3, 4].

AMS interventions have shown effectiveness in meeting medium-to-long-term goals such as decreased consumption of broad-spectrum antimicrobials, cost, and mortality [5, 6]. However, analyses have been limited by heterogeneity of AMS interventions and difficulty inferring causality due to observational study design. There has been less investigation of short-term goals of AMS programs including behaviour change of prescribers. To achieve desired changes in behaviour among prescribers, including greater compliance with antimicrobial guidelines and more appropriate prescribing, psychological theory suggests that there must be antecedent changes in prescribers’ knowledge, perceptions and attitudes towards such behaviour [4].

Annual point prevalence surveys in Australia have indicated differences between private and public hospitals, with private hospitals reporting a higher rate of inappropriate prescribing and non-compliance with antimicrobial guidelines, yet the reasons for this remain poorly understood [7, 8]. To date, Australian studies have mainly evaluated AMS interventions in the public hospital setting, despite 49% of Australian hospitals being private, and similarly mandated to engage in AMS activities, including hospital accreditation processes by national healthcare regulatory bodies [9, 10]. Australian private hospitals have a different organisational structure to the public setting. Medical practitioners are not employees of the hospital but are admitted to practice there, reducing the influence the private hospital manager has over admitting medical practitioners [11]. Therefore, exploring healthcare worker perceptions of AMR and AMS in the private sector is important. Furthermore, few studies have compared knowledge, attitudes and beliefs before and after implementation of an AMS program [12].

In 2013, Cotta et al. undertook focus group discussions and a survey with key AMS stakeholders at a large Australian private hospital as part of a body of work to inform and guide the implementation of an AMS program in the study private hospital [13, 14]. They found that, while less than half of the respondents (45%) believed AMR was a serious problem, the majority did agree with proposed stewardship interventions [14]. Participants highlighted that the autonomy of consultant specialists, peer pressure to conform with other prescriber practices, and a lack of antimicrobial knowledge were all barriers to appropriate antimicrobial prescribing [13]. An AMS program was subsequently implemented at this private hospital in 2014.

We undertook focus group discussions and a survey with key AMS stakeholders at the same private hospital, approximately five years after implementation of the AMS program and Cotta et al.’s pre-implementation analyses [13, 14]. This study aimed to explore capabilities, opportunities and motivations of key AMS stakeholders towards AMR and AMS initiatives at the hospital and compare with the pre-implementation findings. The specific research objectives were to examine the barriers and enablers to implementation of AMS at the hospital in the context of behaviour change.

## Methods

This study was undertaken at the same private hospital as Cotta et al.’s pre-implementation analyses [13, 14]. It employed similar mixed-methods approaches, updated to reflect a post-implementation analysis, and new participants were recruited.

### Setting

**Fig. 1:**
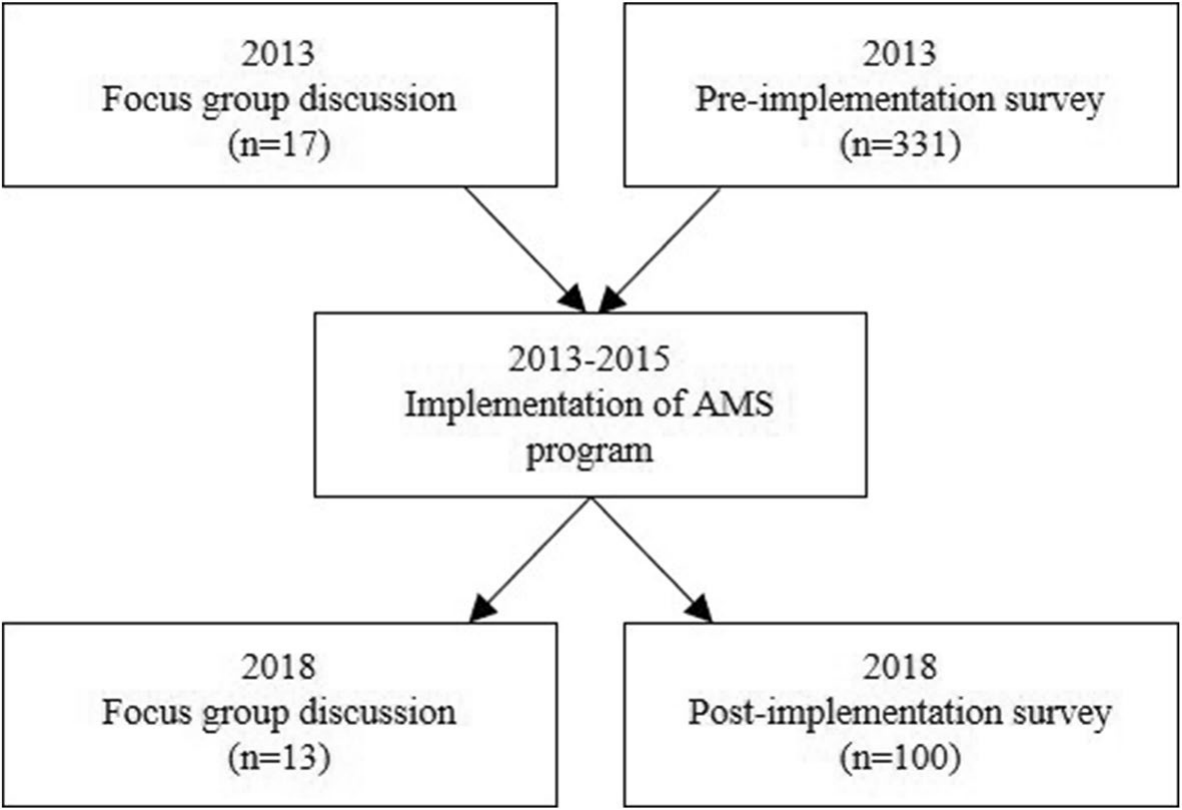
The timeline and overview of participant numbers of the pre-[13, 14] and post-AMS implementation analysis

This was a single-centre mixed methods study conducted at a 766-bed private hospital in Melbourne. The hospital provides a broad range of emergency and elective medical and surgical services, excluding obstetrics and paediatrics. Before the implementation of the AMS program in 2013, pre-implementation studies were conducted which involved a staff survey and focus group discussions (Fig. 1) [13, 14]. Between 2013–2015 the hospital implemented an AMS program, in keeping with mandatory accreditation standards [10]. Key elements of the program included establishment of a governing AMS committee, implementation of formulary restriction processes, development of surgical antimicrobial prophylaxis guidelines and initiation of post-prescription feedback and review by the AMS team, consisting of a full-time senior pharmacist and three infectious diseases physicians. AMS physicians in the study hospital have a fractional appointment. The AMS team provides post-prescription review for broad-spectrum antimicrobials (such as ceftriaxone, piperacillin/tazobactam or meropenem) or high-cost, highly restricted antimicrobials (such as ceftazidime-avibactam) or, in the event of a significant positive blood culture result (such as isolation of *Staphylococcus aureus* in blood cultures). Feedback on the prescription is provided if the antimicrobial therapy deviated from guideline recommendations. AMS implementation at this hospital included direct feedback to prescribers provided by AMS pharmacists and clinicians, and reporting of point prevalence survey results and antibiograms to clinicians and hospital executives.

The program was initially resourced to launch only at the studied hospital, however, it was expanded to two other hospitals in the private hospital group without change to the staff resourcing. Additionally, the size of the study hospital has grown since the program launched leading to further resource spreading. This study was approved by the hospital’s research ethics committee. Invitations to participate in the focus groups and the survey were emailed from executive officers to doctors, nurses and pharmacists who undertake direct or supervised clinical care of patients at the hospital. The hospital has 1090 visiting medical officers, 1200 nurses and 21 pharmacists.

### Focus group discussions

Participants were purposively sampled, having been identified as key stakeholders of the AMS program, following methods employed by Cotta et al. [13]. The focus groups consisted of a range of health professionals as outlined in Table 1, none were AMS team members. Participants who attended signed a consent form.

**Table 1:** Focus group participants

Group 1	One ID physician, two clinical microbiologists
Group 2	One cardiologist, one anaesthetist, one endocrinologist
Group 3	Four nurse unit managers, two hospital executives, one pharmacist

Three focus group discussions were undertaken between December 2018—January 2019. Two researchers (JB & DA) facilitated the discussions using a semi-structured interview guide (Supplementary Material 1) pertaining to participants’ attitudes and perceptions towards AMR and AMS including AMS policies and guidelines. Discussions were audio recorded, deidentified and transcribed verbatim. Data was managed in NVivo™.

### Survey

The 26-item survey (Supplementary Material 2) was based on the pre-implementation survey [14] with some questions altered to reflect post-implementation analysis. The initial survey was developed by an expert, multidisciplinary team consisting of infectious diseases physicians, clinical microbiologists, AMS pharmacists and nurse practitioners [14].

The survey was conducted online using *Qualtrics*™*.* Email invitations contained a link to the survey which was available for 9 weeks from November 2018. Agreement to the introductory statement served as implied consent. Questions were multiple choice or used a Likert scale. Personal information was collected on profession, clinical speciality and years of experience. The survey covered topics including factors contributing to AMR at the hospital, and participants’ awareness of, and agreement with the AMS program at the hospital. All questions referred specifically to the study hospital. Missing or incomplete survey responses were not retained.

### Analysis

Descriptive analysis was undertaken for the survey, with categorical data presented as proportions for each profession. For Likert scale responses, participants were considered “in agreement” or to have viewed AMR as a “serious problem” if they indicated a response of ‘6’ or ‘7’. Pre- [14] and post-implementation response differences were tested using Pearson’s chi-squared test, with a *p*-value < 0.05 considered statistically significant.

Two researchers (EW and JB) independently coded and recoded transcripts using NVivo. Analysis of focus group data was continuous with deductive coding applied from the pre-implementation research, and Michie et al.’s behaviour change wheel [13, 15]. The Behaviour Change Wheel (BCW, also referred to as the COM-B model) was the guiding framework for this research. The BCW consists of three interacting constructs to explain behaviour − capability, opportunity and motivation. Capability is an individual’s knowledge and skills (eg. psychological and physical activity) to engage with the program [15]. Motivation is the reflective and automatic brain action that triggers and directs behaviour (eg. choice and intention) [15]. Opportunity is defined as external factors (social and environmental) that influences behaviour [15]. The BCW has been widely adopted in implementation and health services research. Mapping themes to these conditions allows for an understanding of the areas where an intervention is succeeding or failing to change behaviour. All authors reviewed coding and discrepancies were resolved by discussion.

Survey and focus group data were analysed separately with a process of triangulation applied at the interpretation stage of the analysis to determine whether the findings were convergent, complementary or contradictory [16]. This process involved mapping the survey analysis to the themes identified from the BCW and determining if the qualitative themes were consistent with the survey, whether the qualitative themes expanded or provided nuance to the survey results, or whether the survey and focus group results differed.

## Results

There were 100 responses to the survey in total, including 32 physicians, 15 surgeons, 19 anaesthetists, 21 nurses and 13 pharmacists. Based on the overall staff numbers at the hospital, this represents a response rate of 6% for clinicians, 2% for nurses and 62% for pharmacists. The majority of participants (71%) had > 11 years of experience since their primary healthcare qualification. The survey population had similar years of experience to the pre-implementation survey [14], however, the current study had a higher percentage of physicians and pharmacists, and lower percentages of the other professions (Supplementary Material 3. Supplementary Table 1). Three focus groups involving a total of 13 stakeholders were conducted. Table 1 provides details of the number and profession of participants in each focus group. Themes identified in the COM-B domains are described below in the context of barriers and enablers to implementation of the AMS program. Post-implementation survey findings are presented in comparison with pre-implementation survey results, previously reported by Cotta et al. [14].

## Capability

### Facilitators

Participants had varied antimicrobial knowledge and experience (Table 2), ranging from extensive to little involvement with antimicrobial prescribing and AMS. Participants discussed changes in prescribing habits and number of presentations of AMR organisms over time. The survey results reflected this with 73% of respondents noticing increased AMR presentations in the previous 10 years (Table 3). Similarly, the proportion of survey respondents previously involved in the care of patients with AMR significantly increased (84% to 98%, *p* < 0.0001) from pre- to post-implementation (Table 3). There was a significant increase in awareness of AMS programs from 2013 when only 41% of respondents had heard of AMS (*p* < 0.0001) (Table 3). The clinical microbiologists, infectious diseases physician and pharmacist had the most accurate understanding of the hospital’s program. The clinicians interviewed who were not directly involved in AMS were comfortable knowing when to refer to those with more expertise.

**Table 2:** Capability themes and key quotes

Capability theme	Quotes
Varied antimicrobial knowledge and experience	“[prescribing doctors’] previous experience is very vital… So how often they do use antibiotics. Oncologists have a long history, they’ve got a lot more association with antibiotics than our surgical colleagues, some surgical colleagues still use very old-fashioned approaches to antibiotics and they tend not to keep as up to date as they perhaps should.” (Clinical Microbiologist 1, FG1) “I work in orthopaedics so we have a lot of resistance… even in the last 12 to 24 months we see a lot more coming through… resistance to multiple things and other infections occurring in patients’ wounds.” (NUM 2, FG3) “NUM2: I remember when we first got out first VRE patient on the orthopaedic unit, and it would have been probably 9 or 10 years ago…. [now] it’s kind of like everyone seems to have VRE NUM3: Everyone, I know! … it’s like the boy who cried wolf. It doesn’t shock you anymore.” (FG3) “I know that there are certain antibiotics that I personally shouldn’t prescribe, that are beyond my ability, so I have a low threshold to get an infectious diseases physician involved for anything out of my depth” (Cardiologist, FG2)
Staff understand the theory of AMS	“My understanding of it is… it’s maybe multidisciplinary involving infectious disease physicians, nurses, and pharmacy sort of taking responsibility for appropriate guidelines and overseeing management of antibiotic use in the hospital. That’s my simple view of it.” (Anaesthetist, FG2) “I’m very much aware of the relevance of the problem currently with resistance and lots of different programs either within hospitals or nationally and even internationally to try and reduce said infections that are resistant to standard therapy. I think it’s something that they, I think that even though there is lots of talk about it, the significance is still underplayed.” (Pharmacist, FG3)
Staff have limited knowledge of the hospital’s AMS program	“I think it probably, there would be fair to say there’s some confusion over an infectious diseases specialist seeing someone they’ve been referred to versus an infectious disease doctor doing an AMS round? Like looking at it from a stewardship point of view rather than, from a “I’ve been referred to see a complex patient”.” (Pharmacists, FG3) “You know who [the AMS team] are. But I guess like, you don’t know how they got there. If that makes sense. Were they referred? How do they know? I don’t know that. … Like how do they know to come to bed 54 when like, maybe there’s another 6 patients on antibiotics and you kind of think “oh why are they on vancomycin”.” (NUM 2, FG3) “Do we or don’t we? [laughs] I’m not even sure if we have [an AMS program]” (Cardiologist, FG 2)

**Table 3:** Experience with antimicrobial resistance and AMS

	**Proportion of respondents in 2018** **% (n)**	**Proportion of respondents in 2013** **% (n)**	***p*-value**
Previously involved in care of patient with an antimicrobial resistant infection	98 (95)	84 (254)	< 0.0001
Have noticed an increased number of cases of antimicrobial resistant infections over the past 10 years	73 (61)	70 (174)	0.56
Have heard of AMS	90 (88)	41 (121)	< 0.0001
The study hospital has an AMS program	66.7 (66)	N/A	N/A

analysis included only yes/ no responses, ‘unsure’ excluded

### Barriers

Most participants understood the rationale and general approaches for AMS programs but had limited knowledge of the hospital’s program (Table 2). Almost 90% of survey respondents stated they had heard of AMS, but only 67% were sure the hospital had an AMS program (Table 3). The clinical microbiologists and the Nurse Unit Managers (NUMs) felt professional education for nurses and junior doctors about AMS and antimicrobial prescribing was lacking.

## Motivation

### Facilitators

The survey showed that 60% of respondents agreed that the AMS team at the hospital should continue. However, appreciation and perceived importance of the AMS program differed between participants (Table 4). Some viewed the program as “a very critical part” (CM1, FG1) of infection control in the hospital. However, some were doubtful of the effectiveness of the program, indicating that stewardship does not require “major formalisation” (Endocrinologist, FG2). Participants across the three focus groups agreed on the importance of monitoring and governance of antimicrobial prescribing data to encourage appropriate prescribing and review of technique.

**Table 4:** Motivation themes and key quotes

Motivation theme	Quotes
**Staff have mixed appreciation for AMS within their own practice**	“Well I think there are still a lot of positives. I think it’s a really good program and it’s such an advance on what we had, which was just uncontrolled whatever anyone wanted. So, there’s a lot of positives to it.” (ID physician, FG1) “In the acute casualty area and so on, there may be a role and I don’t know, it depends on the level of training that people come through, ‘cause in our time there was a different training, there were fewer antibiotics, and maybe there is a need for that, but I don’t know.” (Endocrinologist, FG2)
**Lack of staff engagement with the AMS program**	“The therapeutic guidelines … a lot of doctors even now, still don’t even know it exists. … they can find it if they can be bothered looking for it. But people are a bit lazy you know, they aren’t going to do it.” (Clinical microbiologist 2, FG1) “I presume there’s an intranet that [the guidelines] might be available on, but I don’t use it” (Anaesthetist, FG2) “Pharmacist: I don’t know whether people have even seen, but the AMS pharmacist and AMS team put together [a document] which recommends the standard prophylactic surgical stuff and that is on the intranet, and it is a guide that is supposed to be what [the hospital] supports as an organisation, but like you said, there’s no accountability to that document NUM2: I’ve never seen it NUM3: It’s definitely stuck up around theatre, but so is a lot of stuff.” (FG3)
**Staff want to receive more feedback and monitoring data**	“I think there is an unaddressed issue that there’s no monitoring of the ID physicians’ management. So, they’re not actually answerable to each other. … So that’s regarded as success, is referral to an ID physician, and occasionally we’ve had cases where we’ve not agreed with what they’ve done and they’re also not subject to their own internal peer review. And I think that if we had that it would hugely strengthen the whole program” (Clinical microbiologist 1, FG1) “I’d like to be pulled up if I’m doing the wrong thing, but again no one’s ever done that to me.” (Cardiologist, FG2) “We all question as clinicians, oh that’s not right, but the patient still gets it. Cos there’s no … system or processes to say well actually no, that’s inappropriate. Like there’s no sort of big brother” (Executive 1, FG3) “And like you said the standard [antimicrobials] isn’t tracked so you can’t, aside from doing an actual audit, you can’t track the baseline stuff. It’s only the overuse of say Tazocin that we can see because we dispense it. Then we can track it and then the AMS pharmacist can go and say no.” (Pharmacist, FG3) “I think the general antibiotic dispensing is something that needs to be looked at. ‘Cause it’s only sort of the drugs that are like prescribed from dispensing are the ones they look at, whereas it’s the everyday, every joint, that are prescribed.” (NUM 2, FG3)
**Cost is a driving force of AMS and antimicrobial prescribing**	“The other thing that is a factor, from a business model more than anything else, the drugs that are in that category start to become more expensive, so as a private organisation we absolutely care because the fund may or may not pay for it. And they also obviously result in people being in hospital for longer, so not only is the pharmacy cost increased but the actual physical cost of that patient becomes an issue for a department, separate to the whole resistance issue.” (Pharmacist, FG2) “with our patient when she was on the really expensive drugs, there was a lot of debate going on about it and who would pay, there was a lot of cross-questioning done to the infectious diseases doctor and the surgeon. That’s the only time I’ve really seen them question big time, like the medical director was involved, like one patient, ‘cause of the cost.” (NUM2, FG 3) “I think we’re slowly making headway but we’ve still got a way to go. But [improvements to AMS are] all expensive.” (Clinical microbiologist 1, FG1) “we’ve got health funds who are saying, if there’s a hospital acquired infection or a complication we’re not going to pay, because you should have done something about it.” (Executive 1, FG3)

The cost of patient care, antimicrobials and hospital services was highlighted as a “driving force” (NUM 3, FG 3) for the private hospital sector, with participants noting the focus on “getting people out of bed so you can put someone in it” (Pharmacist, FG 3). The high price of some antimicrobials was seen a factor influencing treatment choices, with participants saying the cost can be “very scary” (Executive 1, FG 3) due to concern that private health insurance funds will not cover it. While others noted that the high cost may “improve practice” by encouraging less use of “the scary drugs” (Pharmacist, FG 3).

### Barriers

Staff who had less involvement with antimicrobial prescribing showed little desire to engage with the program, indicating they were unlikely to access guidelines and resources. Surveyed staff believed antimicrobial resistance was significantly less of an issue at the surveyed hospital (40%) compared with hospitals Australia-wide (58%) (*p* < 0.001). The implementation of the AMS program did not have a significant impact on attitudes of respondents, with 62% of respondents from the 2018 survey agreeing that improving antimicrobial prescribing at the hospital would decrease AMR, compared to 58% of respondents in 2013 (*p* = 0.450) (Table 5).

**Table 5:** Responses to statements on patient care, antimicrobial prescribing and antimicrobial resistance

	**Proportion of respondents in 2018** **% (n)**	**Proportion of respondents in 2013** **% (n)**	***p*-value**
Antimicrobial resistance is a serious problem at the study hospital	40 (39)	45 (149)	0.47
Antimicrobial resistance affects patients under my care	44 (41)	36 (119)	0.25
There is antimicrobial prescribing across the study hospital that does not comply with current national antimicrobial guidelines	34 (32)	31 (101)	0.47
Improving antimicrobial prescribing at the study hospital will help decrease antimicrobial resistance at the hospital	62 (58)	58 (192)	0.45

Percentage of respondents ‘in agreement’ (i.e. with a ‘6’ and ‘7’ Likert scale response) (*n*)

Some clinicians in the focus groups expressed concern they may be prescribing incorrectly but had not been approached about it and felt they should be. Staff were not aware of the progress of the AMS program, and felt that receiving feedback could highlight its impact.

## Opportunity

### Facilitators

All three focus groups expressed an opinion that all staff have a responsibility to ensure appropriate antimicrobial prescribing (Table 6). Knowledge and input from specialist staff was valued, with staff frequently referring to the expertise of infectious diseases physicians. The AMS pharmacist was considered a key player in the program, providing valuable input and guidance. The high level of knowledge that nursing staff have of their patients and the wards was also considered important for ensuring appropriate prescribing, and some participants noted that increasing their role in AMS could be beneficial for the success of the program.

**Table 6:** Opportunity themes and key quotes

Opportunity theme	Quotes
**Everyone is responsible for appropriate prescribing and decision making**	“I think all of the medical professionals involved in the patient’s care should be responsible [for appropriate antimicrobial prescribing]. … Everybody. The pharmacist, the surgeons, the ID physicians, the general physicians, the microbiologists, the whole lot and the hospital executive are responsible as well. … Everybody looking after patients need to know when to use antibiotics and which ones to use, for how long, and doses, everybody needs to know that.” (CM 1, FG 1) “And because I don’t admit patients myself, so although I’m responsible for in part of their care … I’m not the decision maker on which antibiotic and unfortunately I sometimes have to give what I don’t necessarily think what is necessary or correct.” (Anaesthetist, FG 2)
**Specialist staff supporting AMS are valued by clinicians**	“Executive 2: we have an antimicrobial stewardship pharmacist… He works closely with the infectious diseases doctors and he tracks and monitors that and reports in a sort of governance way every month … Pharmacist: … the AMS pharmacist does a brilliant job and does have a huge role in intervening, monitoring, and then obviously reporting back” (FG 3) “We’re blessed with very good ID physician cover here, so that no matter what happens you usually get someone involved if you need to for specialist areas.” (Endocrinologist, FG 2) “Nursing staff are really valuable … they’re our allies… they’re a very vital key in that chain because they’re the ones who are on the wards… all the nursing staff see all the patients, so they can highlight something that’s going on.” (CM1, FG1) “Within our departments [nursing staff are] probably the best person on the ward that knows everything that’s going on. So, we can contribute.” (NUM2, FG 3)
**Clinicians value their autonomy**	“No idea what the [hospital] guidelines are. But I’m not changing mine [prescribing practices]. I’m like that surgeon of yours. I haven’t had an infection in 19 years, mine are working.” (Cardiologist, FG 2) “I guess because we’re used to seeing it and the certain people who have a certain preference or a certain way of doing things, whether or not it matches guidelines or not, we sometimes forget to question it.” (Pharmacist, FG 3) “one of the surgeons has his own variation on [the surgical prophylaxis guidelines]. And I’m not the decision-maker for which antibiotic. So, if he wants that, as long as the patient’s not allergic to it or there is no other contraindication, so, a joint replacements, I know cephazolin is the drug we should be using but he adds ceftriaxone” (Anaesthetist, FG 2) “NUM3: A lot of [the doctors], they don’t read [the guidelines]. Even if they do, like we said, they’ve just got their practice. Because it is a culture, no one’s forcing them to do anything. They don’t actually have any rules NUM2: Yeah, they can do what they want. Basically.” (FG3) “Pharmacist: if the prescriber says that “well I’ve done that for 5 years and I’ve never had an infection so why wouldn’t I?” … It’s hard to argue that resistance thing ‘cause they know for them their patients have had a good outcome NUM 2: And it could be related to the antibiotic or it could just be related to their practice Pharmacist: Yeah, the antibiotic might not be necessary.” (FG3)
**Leadership is needed for AMS success**	“You feel like it needs to come from you know, the medical director … we can question as much as we like but I don’t think … it will particularly change their practice.” (NUM 3, FG 3) “If the ICU consultant can’t event ask the surgeon a question, how can the nurses ask them a question?” (NUM 4, FG 3) “It’s kind of like, who governs the prescribing doctors here? … is it the exec or is like the medical director? And if you raise it what kind of response are you going to get? … but there’s not that much governance in the private sector, in my experience. I worked in the public system a really long time ago, but here they can just do what they want.” (NUM 2, FG 3) “I think that’s where sometimes when we talk about being a private organisation, that has lots of visiting doctors… you can’t give them rules but you can give them guides. Where sort of the passive audit approach is potentially helpful to say well, this is the guide and this is what you’re doing – can we talk about it? And that’s about as far as our senior people can actually… question those prescribers that you can’t question… They’re never going to change unless it happens from above, there’s no point the nurses calling or myself calling for that.” (Pharmacist, FG 3) “[The AMS program] has the support of the hospital and the executive because without that it would never be successful, and I think it is very successful here” (Clinical Microbiologist 1, FG 1)

### Barriers

Participants suggested that due to the hospital being private, clinicians have a high level of autonomy in their prescribing choices and frequently did not follow guidelines, instead maintaining their historical prescribing practices. Only 17% of survey respondents frequently consulted the antimicrobial guidelines. Some felt that staff at lower or equal hierarchical levels to prescribing doctors could not question others’ prescribing habits, even if it was against guidelines “because they know they’ll start a fight” (Pharmacist, FG 3). Similarly, one executive (FG 3) stated that there are no processes to empower junior staff to speak up and be protected if they question practice. Participants recognised the importance of the hospital executives’ championship of AMS programs to help drive further system changes, stating that “the only way to fix [antimicrobial prescribing] is from the top” (Pharmacist, FG 3) (Table 6). The survey showed that significantly more respondents were willing to participate in initiatives involving antimicrobial use in 2018 (68%) compared with 2013 (50%) (*p* = 0.003) (Table 7).

**Table 7:** Responses to statements on AMS interventions and willingness to participate

	**Proportion of respondents in 2018** **% (n)**	**Proportion of respondents in 2013** **% (n)**	***p*-value**
I would be willing to participate in any initiatives involving antimicrobial use at the study hospital	68 (63)	50 (167)	0.003
The current antimicrobial prescribing policy at the study hospital should continue	49 (45)	N/A	N/A
The current antimicrobial prescribing restrictions and approval processes in place at the study hospital should continue	50 (46)	N/A	N/A
The AMS team at the study hospital should continue	61 (63)	N/A	N/A

Percentage of respondents ‘in agreement’ (i.e. with a ‘6’ and ‘7’ Likert scale response) (*n*)

## Discussion

AMS programs in the Australian private hospital setting have not been widely analysed. To the best of our knowledge, our study is the first to analyse an AMS program post-implementation in this setting and compare it with pre-implementation data. In 2013, researchers undertook a survey and focus group discussions with key stakeholders at a large Australian private hospital prior to implementation of an AMS program [13, 14]. Our study analysed barriers and enablers to the program’s success five years post-implementation.

### Importance of AMS Staff expertise

The expertise of specialist staff was seen as a benefit for promoting AMS in our study. Appreciation of AMS pharmacists increased post-implementation, and participants had a greater understanding of the importance of their clinical knowledge and guidance than pre-implementation [13]. The benefits of AMS pharmacist input is supported by previous studies [17–20]. Similarly, both our study and the pre-implementation study highlighted the important role that nurses have in AMS due to their contact with patients and clinicians [13]. Nurses are vital in AMS programs through their role in close monitoring of patients, advocating for best patient care and liaising between staff to ensure appropriate antimicrobial prescribing [18, 20–23]. Gillespie et al. [24] demonstrated that increasing AMS education for nurses improves knowledge and may influence antimicrobial management practices. Continued engagement with pharmacists and nurses is necessary for AMS success, and it is vital that their important roles are not overlooked or undermined by hospital hierarchies.

### Staff attitudes towards AMS

While awareness of the AMS program increased, participants expressed doubts about its effectiveness in reducing AMR in the hospital, similar to the pre-implementation survey [14]. The National Antimicrobial Prescribing Survey results for the hospital reflected these results, showing almost no change in rates of appropriate prescribing and compliance with national prescribing guidelines for the five years between 2012–2017 for our study hospital [8].

Participants in our study showed varied understandings of the mechanisms and benefits of the program. Furthermore, staff in both the pre- and post-implementation studies believed that AMS is more of an issue in all Australian hospitals compared with the surveyed hospital [14]. Previous studies have indicated that staff may have difficulty in committing to change actions and behaviours if there is little knowledge of the benefits and outcomes the changes can produce [4, 25, 26]. Other studies have highlighted that this may indicate externalisation of the issue and be a barrier to appropriate antimicrobial management [20, 27–30]. Our results support staff concern about a lack of AMS education, with a shortfall in staff understanding of the importance of AMS in reducing AMR within the institution. Hence staff education may be needed to increase awareness of the AMS program and highlight the benefits.

### Factors supporting behaviour change

Feedback and monitoring of behaviour has been highlighted as a factor in promoting behavioural change [25, 31, 32]. Specifically, feedback and auditing of clinical performance has been shown to improve effectiveness of AMS programs [3, 33]. Participants reinforced this, with several calling for more explicit governance of prescribing practices to improve AMS compliance. Based on the participant discussions, AMS auditing and reporting processes may not have been readily available or accessed by those end-users who would benefit from it. Research has demonstrated that feedback which provides actionable targets, is tailored to the prescribing group, and delivered monthly by a senior colleague is most effective at promoting behaviour change in AMS settings [33–35]. Understanding clinicians’ desired feedback models could improve uptake of AMS programs [35–37]. Some participants stated that they were not being approached regarding their prescribing practices, but felt they should be. At present, feedback on the prescription is only provided in the instance of significant deviation from antimicrobial guideline recommendations. It is therefore possible that these hospital staff were prescribing in keeping with the guidelines and as such, were not contacted by the AMS team. However, studies have shown that providing positive reinforcement for correct behaviour can improve outcomes including appropriate antimicrobial prescribing [38, 39].

### Private hospital structure and AMS

In private hospitals in Australia, medical practitioners are granted clinical responsibilities in the hospital by a Medical Advisory Committee, rather than being employees [11, 40]. The structure of private hospitals encourages clinical autonomy, limiting the ability of hospital managers to directly influence behaviour change because the clinician is not their employee [11]. Additionally, clinicians who choose to work in private hospitals are more inclined to do so because of the increased autonomy and agency over how they treat their patients [41].

Staff in the private sector have previously highlighted difficulties in enforcing AMS guidelines on medical practitioners [20, 42]. Research has shown that familiarity between the AMS team and prescribing clinicians is an important aspect of AMS and providing feedback to clinicians [19, 20]. Creating an environment where the AMS team are well known to all medical practitioners may improve delivery of feedback and uptake of actionable targets. Staff hierarchy is also a common issue in preventing inappropriate antimicrobial prescribing in both private and public hospitals [18, 20, 43]. Clinician autonomy was highlighted as a key theme in our study, with clinicians indicating infrequent referrals to guidelines and continued use of potentially unsupported historical prescribing practices [18, 42]. Studies have found it is common for doctors to be unwilling to change their prescribing practices and be resistant to guidelines, as they can prevent clinical freedom [44–47].

Executive engagement may be needed to drive further system changes across a complex network. Participants in our study indicated that this view remains. The championship of hospital executive to support and promote AMS is a major determinant of success for the implementation of AMS programs [19, 37, 45, 47].

### Costs and antimicrobial prescribing

Cost was seen as an influential factor on antimicrobial prescribing in the private hospital setting. However, similar to previous studies there are mixed views on whether costs have a positive or negative impact on prescribing practices [42]. It is possible that it can lead to over-prescribing of prophylactic antimicrobials to curb any risk of readmission, or it could mean more thought is taken to prescribe the correct antimicrobials and reduce cost [42]. Despite clinicians favouring private practice due to increased autonomy, there are external factors which impact antimicrobial decision making [41, 42].

### Limitations of this study

This study had several limitations. The survey had fewer participants than the pre-implementation study survey [14]. Additionally, the focus groups did not have the same participants as the pre-implementation study and therefore cannot offer a direct comparison of opinions [13]. However, both the survey and focus groups in this study included participants from a range of different healthcare professions and therefore provided an appropriate variety of responses.

## Conclusions

Implementation of AMS programs in private hospitals involves understanding the unique nuances of this setting. This post-implementation study has shown that while there has been a greater awareness of AMS, staff remain sceptical of its benefits and frequently feel pressure to let others’ inappropriate prescribing habits continue. Education to improve understanding of AMS and its local benefits are required, and more must be done to engage medical consultants to be part of the process. To do this, an understanding of their desires for engagement and provision of feedback is necessary, along with the recognised role the executive level play to drive engagement with the program and ensure its success and longevity.

## Supplementary Information

**Additional file 1: Supplementary Material 1.** Semi-structured Interview Guide. **Supplementary Material 2.** Staff Survey. **Supplementary Material 3.** **Supplementary Table 1.** Staff Survey Profession.

## Data Availability

The datasets generated during and analyzed during the current study are not publicly available due to ensure anonymity and privacy of research participants but are available from the corresponding author on reasonable request and with additional ethics approval. Requests for access to data should be submitted via email to A/Prof Trisha Peel (trisha.peel@monash.edu).
