# Supplementary material for: Implementation of an antimicrobial stewardship program in the Australian private hospital system: qualitative study of attitudes to antimicrobial resistance and antimicrobial stewardship
Source: BMC Health Serv Res. 2022 Dec 20;22:1554. doi: 10.1186/s12913-022-08938-8 (PMC9764684; doi:10.1186/s12913-022-08938-8)
Supplement: Supplementary file 1 — Additional file 1: Supplementary Material 1. Semi-structured Interview Guide. Supplementary Material 2. Staff Survey. Supplementary Material 3. Supplementary Table 1. Staff Survey Profession. [file 12913_2022_8938_MOESM1_ESM.docx]

**List of Supplementary Material:**

- Supplementary Material 1 – Semi-structured Interview Guide
- Supplementary Material 2 – Staff Survey
- Supplementary Material 3 – Supplementary Table 1: Staff Survey Profession

**Supplementary Material 1 – Interview Guide**

What is your understanding of antimicrobial resistance or drug-resistant infections?

How does antimicrobial resistance impact on your current practice?

What is your understanding of antimicrobial stewardship?

Can you provide reasons why the [hospital name] may need an ongoing AMS program? Are there any reasons why they might not require an ongoing AMS program?

Do you tend to make use of clinical policies or guidelines relating to antimicrobial prescribing? Why / why not?

What do you think the main challenges are going to be with the continuation of a hospital-wide AMS program at [hospital name] ?

**Supplementary Material 2 – Staff Survey**

**Professional Background**

*What is your profession?*

- Physician
- Surgeon
- Anaesthetist
- Nurse
- Pharmacist

*Medical or surgical specialty (if applicable):*

***_______________________________________________________________________________***

*Years of experience (post primary qualification e.g. post MBBS, BNurs and BPharm)*

- <1 year
- 1 to 5 years
- 6 to 10 years
- 11 to 20 years
- >20 years

**Experiences of antimicrobial resistance and antimicrobial stewardship**

*I have previously been involved in the care of one or more patients with an antimicrobial resistant infection.*

- Yes
- No
- Unsure

*Over the past 10 years I have noticed an increased number of cases of antimicrobial resistant infections.*

- Yes
- No
- Unsure

*I have heard of the term “Antimicrobial Stewardship”.*

- Yes
- No
- Unsure

*‘The study hospital’ has an Antimicrobial Stewardship Program.*

- Yes
- No
- Unsure

**How serious a problem is antimicrobial resistance?**

*Indicate how serious a problem you believe antimicrobial resistance is in the following places (scale 1 to 7).*

|  | 1 (Not a problem) | 2 | 3 | 4 | 5 | 6 | 7 (Very serious problem) |
| --- | --- | --- | --- | --- | --- | --- | --- |
| Worldwide |  |  |  |  |  |  |  |
| Australian Community |  |  |  |  |  |  |  |
| Australian Hospitals |  |  |  |  |  |  |  |
| ‘The study hospital’ |  |  |  |  |  |  |  |

**What contributes to antimicrobial resistance?**

*Indicate how strongly you believe the following contribute to antimicrobial resistance at ‘the study hospital’ (scale 1 to 7).*

|  | 1 (Dose not contribute) | 2 | 3 | 4 | 5 | 6 | 7 (Strongly contributes) |
| --- | --- | --- | --- | --- | --- | --- | --- |
| Use of antimicrobial in the Australian animal/agricultural sectors |  |  |  |  |  |  |  |
| Use of antimicrobials in the Australian community |  |  |  |  |  |  |  |
| Use of antimicrobials in Australian hospitals |  |  |  |  |  |  |  |
| Use of antimicrobials at ‘the study hospital’ |  |  |  |  |  |  |  |

**Antimicrobial resistance at ‘the study hospital’.**

*Indicate how strongly you agree or disagree with the statements below (scale of 1 to 7).*

|  | 1 (Strongly disagree) | 2 | 3 | 4 | 5 | 6 | 7 (Strongly agree) |
| --- | --- | --- | --- | --- | --- | --- | --- |
| Antimicrobial resistance affects patients under my care at ‘the study hospital’. |  |  |  |  |  |  |  |
| There is antimicrobial prescribing across ‘the study hospital’ that does not comply with current national antimicrobial guidelines (ie. Therapeutic Guidelines: Antibiotic). |  |  |  |  |  |  |  |
| Improving antimicrobial prescribing at ‘the study hospital’ will help decrease antimicrobial resistance at the hospital. |  |  |  |  |  |  |  |
| I would be willing to participate in any initiatives involving antimicrobial use at ‘the study hospital’. |  |  |  |  |  |  |  |

**Antimicrobial prescribing at ‘the study hospital’**

*Indicate how strongly you agree or disagree with the statements below (scale of 1 to 7).*

|  | 1 (Strongly disagree) | 2 | 3 | 4 | 5 | 6 | 7 (Strongly agree) |
| --- | --- | --- | --- | --- | --- | --- | --- |
| The ‘current hospital’ antimicrobial prescribing policy should continue. |  |  |  |  |  |  |  |
| The current antimicrobial prescribing restrictions and approval process at ‘the study hospital’ should continue. |  |  |  |  |  |  |  |
| The computer application ‘Guidance-MS’ (used to monitor prescriptions of certain antimicrobials) should continue to be used at ‘the study hospital’. |  |  |  |  |  |  |  |
| The Antimicrobial Stewardship team, consisting of a specialist physician and pharmacist who provide individualised antimicrobial prescribing advice and feedback, should continue at ‘the study hospital’. |  |  |  |  |  |  |  |

**Compliance with antimicrobial guidelines**

*I would estimate that the percentage of INPATIENT antimicrobial orders that do not comply with current antimicrobial guidelines (i.e. Therapeutic Guidelines: Antibiotic) at ‘the study hospital’ is:*

- Less than 50%
- 50% of greater

*I would estimate that the percentage of SURGICAL PROPHYLAXIS orders that do not comply with current antimicrobial guidelines (i.e. Therapeutic Guidelines: Antibiotic or local guidelines) at ‘the study hospital’ is:*

- Less than 50%
- 50% of greater

*For what proportion of antimicrobial prescriptions do you consult antimicrobial guidelines (ie. Therapeutic Guidelines: Antibiotic or local guidelines)?*

- <20%
- 20-40%
- 40-60%
- 60-80%
- >80%

**Additional comments**

*Additional comments (if no additional comments, please click the arrow):*

***__________________________________________________________________________________***

**Supplementary Material 3**

**Supplementary Table 1.**

**Staff survey profession: pre-implementation (2013) and post-implementation (2018) studies, n (%)**

|  | Physician | Surgeon | Anaesthetist | Nurse | Pharmacist | Total |
| --- | --- | --- | --- | --- | --- | --- |
| 2013 | 80 (24.17) | 58 (17.52) | 78 (23.56) | 105 (31.72) | 10 (3.02) | 331 |
| 2018 | 32 (32) | 15 (15) | 19 (19) | 21 (21) | 13 (13) | 100 |

**Staff survey experience: pre-implementation (2013) and post-implementation (2018) studies, n (%)**

|  | <1 year | 1 to 5 years | 6 to 10 years | 11 to 20 years | >20 years | Total |
| --- | --- | --- | --- | --- | --- | --- |
| 2013 | 5 (1.51) | 25 (7.55) | 43 (12.99) | 112 (33.84) | 146 (44.12) | 331 |
| 2018 | 1 (1) | 8 (8) | 20 (20) | 28 (28) | 43 (43) | 100 |
